# Synergic fabrication of succimer coated titanium dioxide nanomaterials delivery for *in vitro* proliferation and *in vivo* examination on human aortic endothelial cells

**DOI:** 10.1080/10717544.2021.1960925

**Published:** 2021-09-02

**Authors:** Ming Qi, Chunfang Li, Ze Song, Lei Wang

**Affiliations:** a Department of Vascular Surgery, The First Affiliated Hospital of Dalian Medical University, Dalian, China; b Department of Nursing, The First Affiliated Hospital of Dalian Medical University, Dalian, China

**Keywords:** Succimer, titanium dioxide, endocytosis, metastasis, chick chorioallantoic membrane

## Abstract

The probable nanotoxicity to human health and the environment is a significant challenge for the sustainable application of nanomaterials in medicine. The cytototoxical effect of succimer (meso-2,3-dimercaptosuccinic acid-DMSA) coated titanium dioxide (DMSA-TiO_2_) with cultured human aortic endothelial cells (HAoECs) was assessed in this investigation. Our findings have shown that DMSA-TiO_2_ can be accumulated in HAoECs and dispersed in a cytoplasm on the culture medium. DMSA-cytotoxicity TiO_2_ effects were dose-responsive, and the concentrations were of little toxicity, and MTT stain testing showed that they had only 0.02 mg ml^−1^. Meanwhile, the lactate dehydrogenase biomarker was not considerably more remarkable than the biomarker from untreated (control) cells (free DMSA-TiO_2_). Though, also without any apparent signs of cell damage, the endocrine functions for prostacyclin I-2 and endothelin-1 and the urea transporter functions were modified. In addition, *in vitro* endothelial tube development has been shown that HAoECs could induce angiogenesis even with small amounts of DMSA-TiO_2_ (0.01 and 0.02 mg ml^−1^). Further, we have examined the *in vivo* toxicity and biochemical parameter by animal model. Furthermore, *in vivo* assessments designated that the resulting DMSA-TiO_2_ presented synergistic activities of angiogenesis activity. Overall, these findings show the cytotoxicity of DMSA-TiO_2_ and could induce adverse effects on normal endothelial cells.

## 1. Introduction

The use of Nanomaterials for drug delivery applications, antimicrobial materials, cosmetics, sunshades, and electronics has increased dramatically with the advancement of nanotechnology (Aranega and Boulaiz, 2005; Delplace et al., 2014; Huang et al., 2017). In October 2019, the European Union specified nanomaterials as the unbound or aggregate or agglomeration, natural, incidental to or produced particle material; in which 50% or more exhibited particles one or several external dimensions ranging from 1 to 100 nm (Duo et al., 2017; Llinàs et al., 2018; Tambe et al., 2018). Others characterized nanomaterials as objects that range from 1 to 100 nm in at most one among their three dimensions. The physicochemical characteristics of Nanomaterials usually are significantly different from the fine particles (FPs) of the same structure (Shen et al., 2011; Kim et al., 2015; Zhu et al., 2016). The smaller scale of nanomaterials ensures a significant portion of atoms on the particulate surface. As the subfactors, including electronics, reactivity, and energy levels, vary significantly from internal conditions, nanomaterials bioactivity is likely to differ from the perfect analog size (Dahl et al., 2014).

Titanium dioxide nanomaterials (TiO_2_ NMs) have traditionally been considered low-toxicity particles that are poorly soluble (Skuza et al., 2016; Johnson et al., 2017; Ali et al., 2018). Therefore, in many *in vitro* and *in vivo* component toxicology investigations, they were typically used as a ‘negative control.’ However, this is a challenge after the development of tumors in rats following two years of exposure to elevated TiO_2_ rates (Raza et al., 2021). Therefore, TiO_2_ has been categorized as a Group 2B cancer by the International Agency for Cancer Research (IARC). However, instead of being particularly carcinogenic in fine TiO_2_, the tumorigenic impact of fine TiO_2_ was questioned and attributable to lung overload. Due to their high catalytic action compared to TiO_2_ NMs, TiO_2_ NMs have been widely used in many industrial and consumer products in recent years (Uk Lee et al., 2015; Gupta et al., 2016; Kubo-Irie et al., 2016; Bejarano et al., 2018). Their smaller sizes have been the reason for this increase in catalytic activity, enabling more extensive surfaces per unit mass. There is concern that the same TiO_2_ NMs properties can pose specific bioactivity and human health challenges (Lucky et al., 2016). The rapid rise in the number of reported studies shows that the protection of TiO_2_ NMs is highly concerned. Numerous animal models, including inhalation, dermal, intratracheal, oral gavage, intraperitoneally, or intravenous injections, have been rigorously used in these trials (Guo et al., 2020; Mei et al., 2020; Zhao et al., 2020).

In terms of being able to inject a large number of TiO_2_ NMs into vessels that are endothelial cells (ECs) lined by one epithelial scalp and anticontainous membrane between vessel wall and blood, many biological properties of TiO_2_ NMs, including magnetic detection and hyperthermia, require a great deal (Dedman et al., 2021; Escudero et al., 2021; Liang et al., 2021). The ECs is a modulative agent for blood flow and blood vessel sound, which contributes to inflammatory and immune response, coagulation, growth controls, extracellular matrix's formation, and ECs damage, activation, or impairment are characteristic of specific disease conditions, such as atherosclerosis, lack of semi-permeableness and thrombosis (Zhang et al., 2021). A large number of stimuli can cause endothelial cells to die from their programmed cellular (apoptosis) through their extrinsic (death receptor), and apoptotic pathways (mitochondria), which are carried out through caspases called the intracellular proteases (Liu and Chen, 2014; Katir et al., 2019; Yang et al., 2021). The mechanisms of cell death and anti-apoptotic proteins are also caspases-independent and can shield cells from apoptosis. The complicated cell apoptosis network consists of these pathways and proteins. ECs are the first tissue obstruction experienced by TiO_2_ when inserting TiO_2_ NMs into blood vessels (Fattakhova-Rohlfing et al., 2014). This study aims to evaluate cytotoxicity in human aortic endothelial cells (HAoECs) for DMSA-coated TiO_2_ nanoparticles. These nanoparticles proliferate over many generations while retaining their endothelial characteristics and are widely utilized *in vitro* and *in vivo* studies.

## 2. Experimental section

### 2.1. Materials

Titanium dioxide (TiO_2_) was purchased from China Petrochemical Group Co., Ltd., China. Dulbecco's Modified Eagle's Medium (DMEM), fetal bovine serum (FBS), 3-(4,5-dimethylthiazol-2-yl)-2,5-diphenyltetrazolium bromide (MTT), penicillin-streptomycin, and trypsin were brought from Invitrogen. Other reagents and solvents are of analytical grade and used without any purification.

### 2.2. Preparation of DMSA-TiO_2_ nanoparticles

The previously reported protocol prepared TiO_2_ nanoparticles. Firstly, 0.250 g TiO_2_ substances were mixed in 0.50 ml ethanolic solution with the addition of followed 0.50 ml of DMSA and 0.50 ml acetic acid. The reaction mixture was immersed in the preparation of mortar until of TiO_2_ slurry. According to the process described in the literature, TiO_2_ was coated with DMSA. Finally, stable aqueous sol DMSA-TiO_2_ was obtained. The resulting DMSA-TiO_2_ was sealed at 150 °C for 10 h in a Teflon-lined autoclave. Then, the white precipitates were washed, collected, and air-dried as per the earlier described method (Bai et al., 2014).

### 2.3. Characterization and cell culture

High-resolution Transmission electron microscopy was adopted to characterize as-synthesized DMSA-coated TiO_2_ morphology and crystalline nature (HRTEM) (model Tecnai G2 20 TWIN). Zetasizer Nano ZS (Malvern, UK) successfully obtained dynamic light scattering (DLS) and ζ-potential data. Powder XRD patterns of the DMSA-TiO_2_ were examined by a Rigaku Ultima IV diffractometer operating at 35 kV, 15 mA with CuKα radiation wavelength of *λ* = 1.5406 Å. HAoECs (human aortic endothelial cells, ATCC) were obtained from the Cell Bank of the Chinese Academy of Sciences, Shanghai, China. HAoECs were cultured in DMEM (Gibco, USA) with 10% fetal bovine serum and 1% of streptomycin and penicillin addition at 37 °C in humidified air containing 5% CO_2_.

### 2.4. Assessment of HAoECs location of DMSA-TiO_2_

HAoECs were washed with Phosphate buffer solution (PBS) and regularly immovable, dried out, and implanted in the TEM experiments for 24 h with 0.02 mg ml^−1^ of DMSA-TiO_2_. The TEM samples were prepared by dripping DMSA-TiO_2_ dispersion onto a holey carbon film followed by drying.

### 2.5. Examination of cytotoxicity and cell viability

The tetrazolium dye (MTT) test examined the cytotoxic effects of DMSA-TiO_2_ against HaoECs (Mohamed Subarkhan et al., 2016; Subarkhan and Ramesh, 2016; Mohamed Kasim et al., 2018; Mohan et al., 2018; Balaji et al., 2020; Sathiya Kamatchi et al., 2020). The HAoECs were used for 4, 24, 48, and 72 h for a time-dependence development (0.05 mg ml^−1^) of DMSA-TiO_2_. The DMSA-TiO_2_ has added 24 h in HAoECs to the dose dependence effect, diluted with cultivable medium, at a graduated concentration (0.001–0.2 mg ml^−1^). The HAoECs were incubated with MTT solutions at 37 °C for 1 h after washing with PBS, and dimethyl sulfoxide (DMSO) was dissolved for 15 min. The absorbance values of formazan formed in cytotoxicity assays were measured with a Thermo Varioskan Flash at 525 nm was examined, and cell viability was measured as a proportion of control cells processed free DMSA-TiO_2_. These procedures were repeated three times.

### 2.6. Examination of HAoECs endocrine factors and injury markers

HAoECs have been cultured with 0.02 mg ml^−1^ of DMSA-TiO_2_ for 24 h in this experiment. At 7000 μg, for 30 min at 4 °C to eliminate the remaining nanomaterial and cells waste, the cell culture supernatants were centrifuged. NO, PGI-2, and ET-1 concentrations were assessed using the ELISA kits, respectively, according to the manufacturer's instructions. Automatic biochemistry analyzers were used to detect urea and lactate dehydrogenase (LDH) (LCSM, Olympus Fluoview 1000, Japan).

### 2.7. Examination of capillary tube formations

The tube formations analysis is among the supreme studies utilized for modeling angiogenesis *in vitro* re-organization. The defined as the study the endothelial cell ability to form capillary structures, plated in subconfluent concentrations with the required extracellular matrix supports. The extracellular matrix support used for Matrigel basement membranes matrix has been needed to determine whether angiogenesis of DMSA-TiO_2_ will intervene in the HAoECs. 60 μl/each wells of the membrane matrixes were added to the A96 culture plate to formulate HAoECs tube, and 60 min gel was allowed at 37 °C for 60 min. HAoECs were seeded in the presence or absence of established DMSA-TiO_2_ (0.01 and 0.02 mg/ml) and Urea 6 M at 1 × 105 cells/each well on the surface of the gel then incubated in a CO_2_ incubator for 14 h at 37 °C. 0.1% DMSO was used as a control. In the meantime, the high urea (6 M urea) solution has been added as a positive control for tube forming inhibition. Glutaraldehyde was washed and stained with Mayer's hematoxylin for 10 min in 25% of the cultures. These procedures were repeated three times (Bhagwat et al., 2001; Komorowski et al., 2006; Matsuo et al., 2007).

### 2.8. Examination of cell invasion

50 μl/each well of the membrane matrixes were added to the A96 culture plate for the cell invasion and complete gelation for 1 h at 37 °C. HAoECs were seeded in the presence or absence of established DMSA-TiO_2_ (0.01 and 0.02 mg/ml), along with Urea 6 M at 1 × 1045 cells/each well surface of the gel then incubated in a CO_2_ incubator for 14 h at 37 °C. 0.1% DMSO was used as a control. In the meantime, the high urea (6 M urea) solution has been added as a positive control for tube forming inhibition. Glutaraldehyde was washed and stained with Mayer's hematoxylin for 10 min in 25% of the cultures. These procedures were repeated three times (Hendel and Granville, 2013; Michelini et al., 2016; Menezes et al., 2018).

### 2.9. Examination of chick chorioallantoic membrane

DMSA-TiO_2_ was evaluated *in vivo* with a chick chorioallantoic membrane (CAM) model for its antiangiogenic activity (Rovithi et al., 2017; Vu et al., 2018; Pawlikowska et al., 2020). Briefly, ethanol was used to cleanse the surface of the fertilized chicken eggs, and the incubator was used to incubate the eggs in a humanized 60% atmosphere at 37 °C. After 3 days of incubation on the broad side of the shell with a scissor, a 1 cm^2^ gap was opened, and the membrane was sterilely separated from the CAM tissue. On the 8th day, the sterile filtering paper (5 mm in diameter) was soaked in DMSA-TiO_2_ (0.01 and 0.02 mg/ml) and Urea 6 M solutions for 1 min and then covered into CAM tissue model exposed vessels. As a guide control, saline was used. The CAM tissues were examined after 24 h exposure to filter paper, and at least three random fields were covered with the branched number of vessels. These procedures were repeated three times.

### 2.10. Animals models

ICR mice were purchased from the animal center at Shanghai China Medical University for organ toxicity studies (ICR) mice (22 ± 3 g, half-male, and half-female). All animal testing procedures were pre-approved and performed in compliance with international standards for treating and using research animals by the First Affiliated Hospital of Dalian Medical University (2019-2158/45). Per mouse was sexually ripe and well. In an animal house with adequate ventilation, 12 h light/dark period, 20 ± 2 °C, 60% relative moisture, and *ad libitum* access to food and drink, mice were raised in separate cages five days before the procedure.

Randomly, the mice were split into six groups and an extra 10-mice/group control group. DMSA-TiO_2_ nanomaterials have been injected once daily for 14 days [intraperitoneal (i.p.), 5, 10, 15, 20, and 25 mg/kg]. Saline was pumped into test group mice. The Saline group was used as the control. Every day, the mouse was examined, and during the study, no animal died. Blood samples from the orbital sinus were obtained on the 15th day. Each mouse was weighed individually with a 2% phenobarbital anesthetic (60 ml/kg, i.p.), then sacrificed by cervical dislocation. Two sections have been cut to each heart, liver, spleen, lung, and renal. For pathological analysis, one part was soaked in formaldehyde (10%) solution at 4 °C. To determine the titanium material, the other component was deposited at −20 °C.

### 2.10. Statistical analysis

All of the results are expressed as mean ± *SD*. The statistical significance was performed with Graph pad Prism software using ANOVA. *p* < .05 was considered statistically significant.

## 3. Results and discussion

### 3.1. Structural characterization

**Figure 1.**
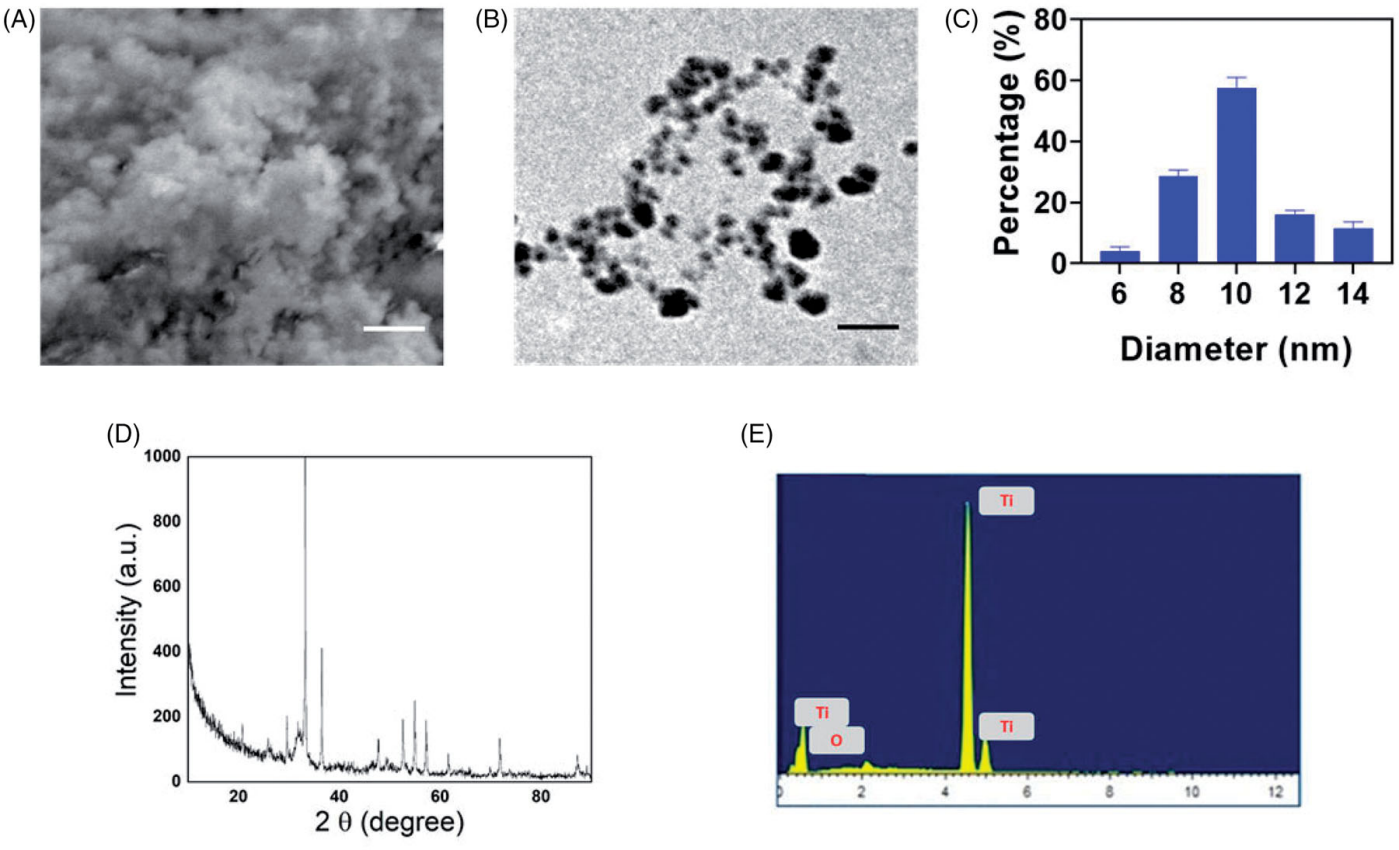
Structural characterization of DMSA-TiO_2_. (A) SEM image of DMSA-TiO_2_. Scale bar 100 µm. (B) TEM image of DMSA-TiO_2_. Scale bar 50 nm. (C) Hydrodynamic parameter of DMSA-TiO_2_ examined by light scattering (DLS) methods. (D) Powder-XRD pattern of DMSA-TiO_2_. (F) Elemental mapping analysis (EDX) images of DMSA-TiO_2_. EDX data reveals that the formation of DMSA-TiO_2_.

The SEM image of the DMSA-TiO_2_, as shown in Figure 1(A), also indicates a similar pattern in the accumulation of spherical particles. EDS spectrums were recorded and displayed in Figure 1(F). DMSA-TiO_2_ has confirmed the presence of the elementary compositions of Ti and O peaks. The figure shows the TEM images of the sol-gel phase of DMSA-TiO_2_. The homogenous distribution of nanoparticles of spherical forms of DMSA-TiO_2_ from lower to higher resolution can be observed in Figure 1(B). The average particle size was ∼12 nm, coinciding with the crystalline structure size determined by the dynamic light scattering (DLS) analysis (Figure 1(C)). Further zeta potential results demonstrate the negative values of DMSA-TiO_2_ (data not shown).

The XRD analysis examines the crystal structure and phase composition of the formulated powder samples. Figure 1(D) shows DMSA-TiO_2_ Powder-XRD patterns observed within the 20–80° range. The pure anatase phases with dominant peaks at 25 and 48° exhibit Figure 1(D) without any contaminants existing. The diffraction peaks are consistent with the anatase process reflections and are indexed using match software in compliance with JSPDS File No. 96-152-6932. The indexed planes support TiO_2_ pure trigonal planar and tetrahedral coordination geometry.

### 3.2. Haoecs endocytosis of DMSA-TiO_2_

**Figure 2.**
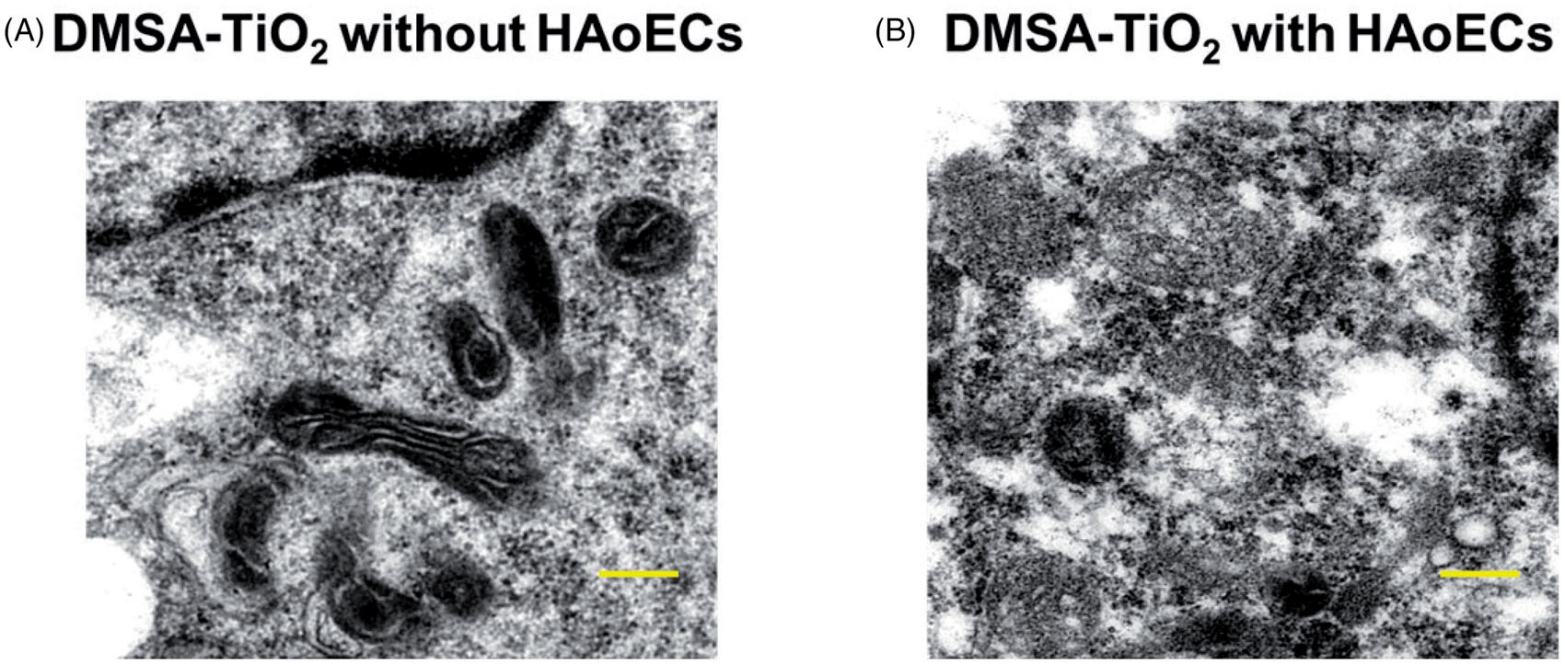
TEM imaging analysis of HAoECs with 0.02 mg ml^−1^ of DMSA-TiO_2_ for 24 h. (A) DMSA-TiO_2_ without HAoECs. (B) DMSA-TiO_2_ with HAoECs. Scale bar (×3000).

In HAoECs, the DMSA-TiO_2_ is recognized and distinguished by high electron density at TEM from the cellular structures (Figure 2). Figure 2 depicts micrographic TEM pictures between 0.02 mg ml^−1^ of DMSA-TiO_2_ incubation and DMSA-TiO_2_ incubations-free HAOECs (Figure 2). Findings demonstrate that the DMSA-TiO_2_ aggregates are easy to absorb and disperse within the cytoplasm by the cells without disrupting the cellular membrane.

### 3.3. Haoecs viability studies

The formazan assay (MTT) has been utilized to detect the amount of live (proliferation) and cell viability (cytotoxic) cells the result of TiO_2_ materials, because the formazan, which can be calculated in terms of quantitative measurements after dissolution in DMSO with the resulting value, can only be reduced by living cells to their insoluble form.

**Figure 3.**
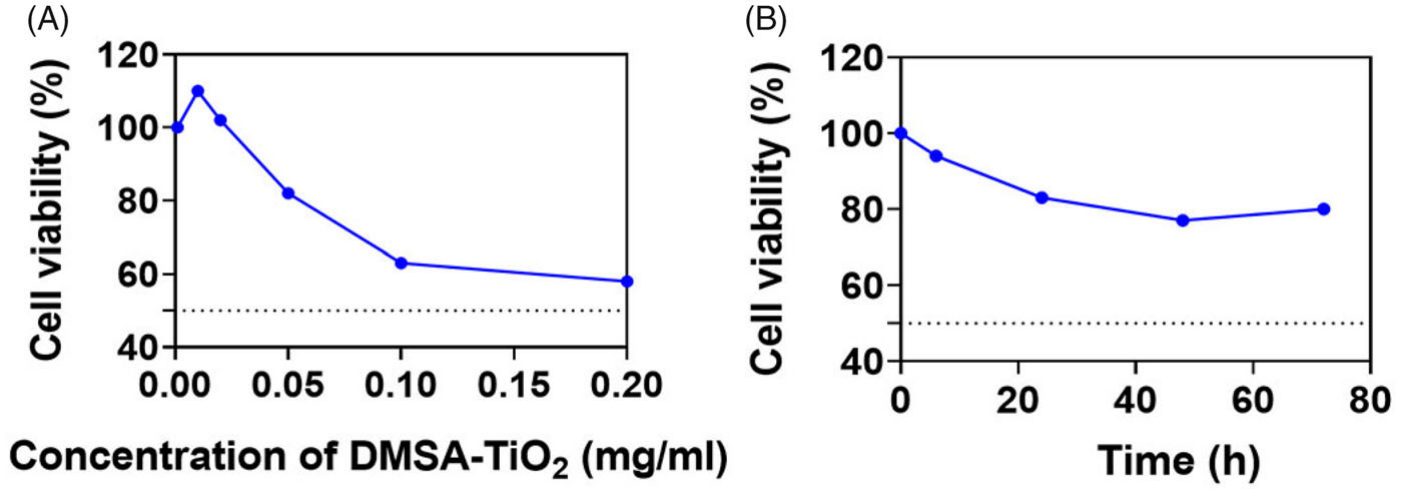
The cell viability examination of HAoECs incubation with DMSA-TiO_2_. (A) HAoECs was incubate with DMEM medium featuring increasing concentrations (0.001, 0.01, 0.02, 0.05, 0.1, 0.2 mg ml^−1^) of the DMSA-TiO_2_ for 24 h. (B) HAoECs was incubate with DMEM medium featuring concentration (0.05 mg ml^−1^) of the DMSA-TiO_2_ for 24 h.

The viability of HAoECs was reduced in the present study compared to that of control cells with enhanced DMSA-TiO_2_ concentration (Figure 3(A)). The 24 h induced no cell losses, HAoECs treated at levels below 0.05 mg ml^−1^ DMSA-TiO_2_. By contrast, DMSA-TiO_2_ was significantly cytotoxic at higher doses (>0.05 mg ml^−1^). At a concentration of 0.2 mg ml^−1^, the cell viability of HAoECs incubated with DMSA-TiO_2_ decreased by ∼60% of the control cells.

HAoECs cells of 0.05 mg ml^−1^ with DMSA-TiO_2_ were incubated with the 4, 24, 48, and 72 h, respectively (Figure 3(B)) to investigate the time-dependent effects of DMSA-TiO_2_ (0.01 and 0.02 mg/ml) and along with Urea 6 M on HAoECs viability. The reduced cell viability in the tested time group ranged by 4 h, ranging from ∼75 to 95%. The findings indicate that the cytotoxic effect on HAoECs of DMSA-TiO_2_ is dose-dependent, and in the current study, the concentrations are not exceeding 0.02 mg ml^−1^, relatively non-harmful.

### 3.4. Effects of DMSA-TiO_2_ on HAOECS injury markers and endocrine factors

**Figure 4.**
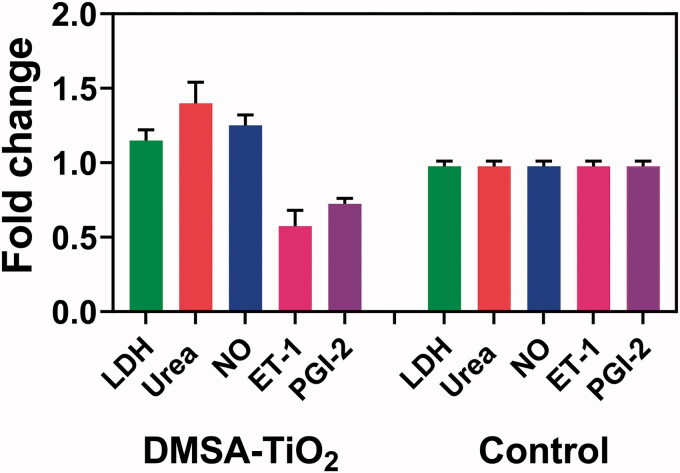
The ratio of DMSA-TiO_2_ on HAoECs injury markers and endocrine factors. HAoECs incubate with 0.02 mg ml^−1^ of DMSA-TiO_2_ (0.01 and 0.02 mg/ml) and along with Urea 6 M for 24 h. Percentages are relative to the control (untreated) cells (free DMSA-TiO_2_).

The LDH enzyme is a cytoplasm that may be released into the extracellular space due to disruptions in cell integrity caused by pathological conditions. Supernatant LDH in HAoECs cultured materials is thus detected as a cell damage marker. We observed that LDH differences were not present from the 24 h with HAoECs incubation and control cells (Figure 4). LDH is 0.02 mg ml^−1^ DMSA-TiO_2_ (0.01 and 0.02 mg/ml) and along with Urea 6 M. The results from the low cytotoxicity effect in MTT (Figure 4), as well as improvements in cell membrane integrity in TEM (Figure 4), were similar to these findings.

We then investigated whether other endocrine functions of HAoECs were changed in the absence of detectable cell injury when exposed to this low dose of DMSA-TiO_2_. Blood pressure and blood supply can be controlled with ECs by releasing NO and PGI-2 vasodilator and ET-1 vasoconstrictors. Thus, by finding out the previous section factors in the supernatant, the endocrine activity of cultured HAoECs can be assessed. In the HAOECs treated with 0.02 mg ml^−1^ of DMSA-TiO_2_ for 24 h, we found that NO's release was not modified (Figure 4). The most effective stimulator for vascular dilator and effective inhibitor to the aggregation and conformity of platelets is NO released into the vascular lumen. NO is one of the essential defensive molecules in a vasculature to prevent the beginning and further steps of atherogenesis. The endothelial NO synthase (eNOS) in the vasculature, the primary responsible vascular NO production, is the prevalent NOS isoform. An eNOS oxidizes the L-arginine base into the L-citrulline with NO substrates. In the HAoEC, eNOS activity is not impaired by 24 h DMSA-TiO_2_ treatment with 0.02 mg ml^−1^.

The HAoECs were treated with 0.02 mg ml^−1 ^h for 24 h (Figure 3). Unlike NO release, the release of another PGI-2 vasodilator and vasoconstrictor ET-1 was significantly reduced. In addition to its efficient vasodilator function, PGI-2 can prevent the forming of platelets by disrupting plates. The action of the PGI-2 enzyme is developed by the activity of the PGI-2 synthase in endothelial prostaglandin H2 cells. ET-1 is constitutively selected by the action of an endothelial enzyme present on both the EC surface and on intracellular vesicles by endothelial cells from the inactive medium major ET-1. Complex signals control the expression and release of PGI-2 and ET-1 on the ECs; the process for their reduction and release was not studied in this study. However, our findings show that HAoECs endocrine functions are sensitive to DMSA-TiO_2_ and may intervene before major cell injuries occur.

We have investigated the cellular uptake mechanism by studying the function of the urea conveyor system and the cell release function of these vessel's tone regulators. The urea transporter is represented in the vascular endothelium that delivers Urea to the cell. In the endothelial cells, Urea plays a key role; previous studies have shown that L-arginine transportation in cultured endothelial cells is inhibited by uremic Urea (25 mM). In this analysis, we find a significant urea concentration higher than that of control cells in the HAoECs, treated with 0.02 mg ml^−1^ of DMSA-TiO_2_ for 24 h. This analysis shows that DMSA-TiO_2_ exposure also inhibits the role of the urea carrier in HAoECs.

### 3.5. Effects of DMSA-TiO_2_ on HAoECs tube formation

**Figure 5.**
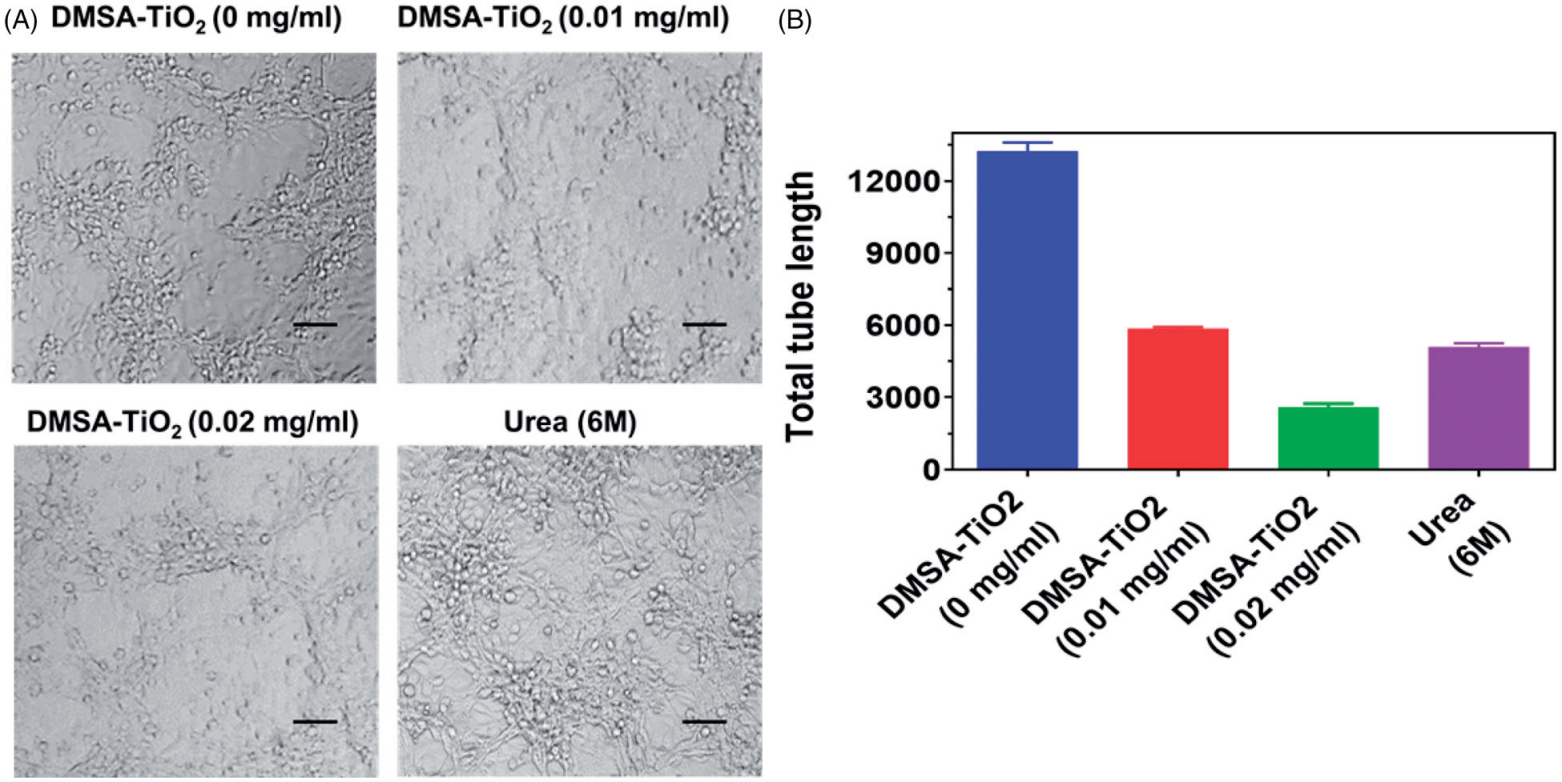
(A) Representative images of tube formation inhibition by DMSA-TiO_2_ (0.01 and 0.02 mg/ml) and along with Urea 6 M on HAoECs. (B) Tube formation capacity of HAoECs. Scale bar 200 µm.

DMSA-TiO_2_ is a promising, anti-proliferative, and cytotoxic vascular disrupting agent. We selected HAoECs that are active in developing a tumor vessel and are a helpful model for *in-vitro* angiogenesis studies to assess if DMSA-TiO_2_ is still active. Diverse analyses have been done to confirm the DMSA-TiO_2_ effects by exposing them to HAoECs, which increase. DMSA-TiO_2_ concentrations used in these analyses are low to reduce the cytotoxic effects within the nanomolar range. HAoECs migration was significantly decreased in wound-healing assays when DMSA-TiO_2_ (0.01 and 0.02 mg/ml) and along with Urea 6 M was treated 24 h a day (Figure 5(A,B)).

**Figure 6.**
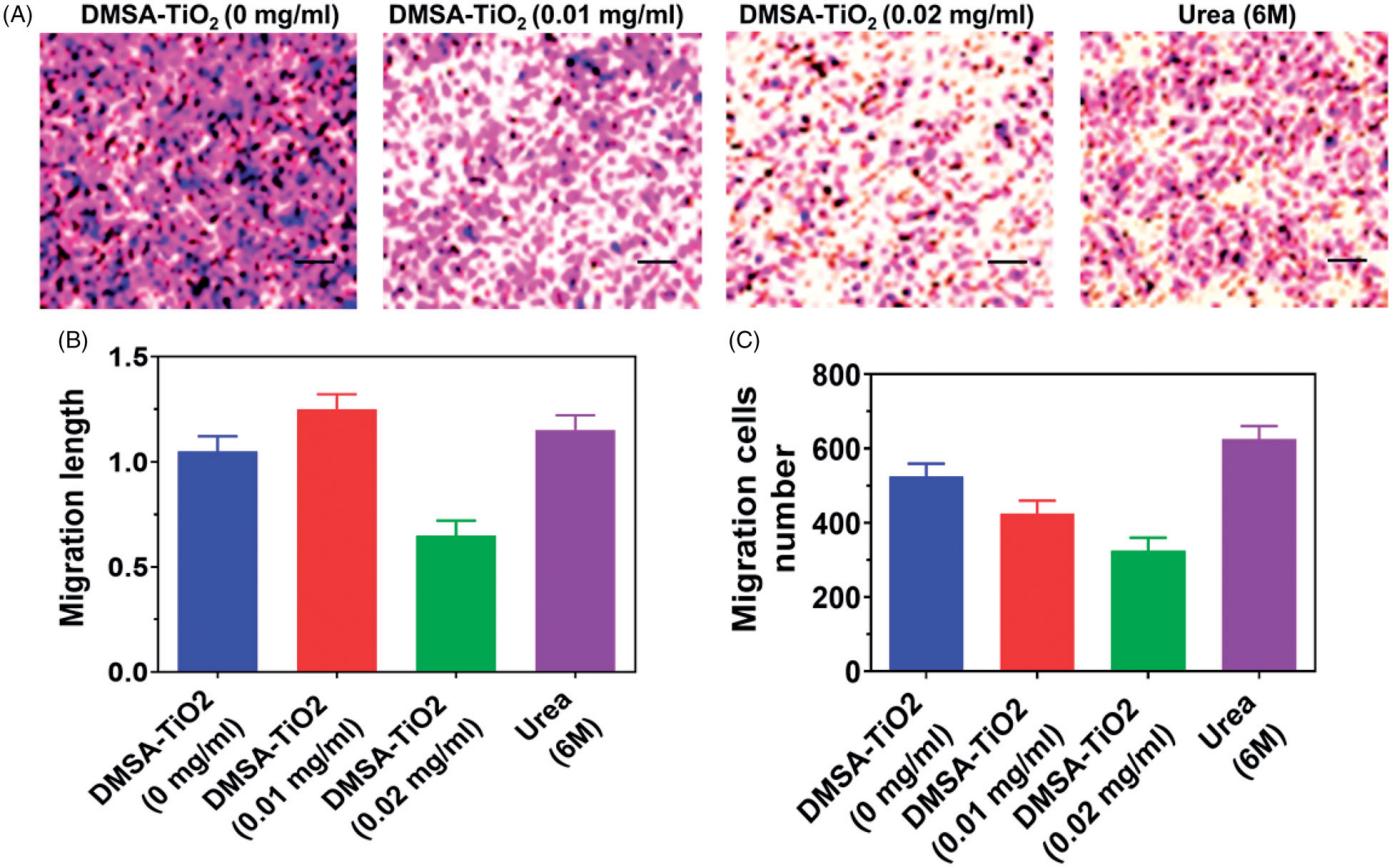
Invasion inhibition by DMSA-TiO_2_ on HAoECs in Transwell assays. (A) Representative images of HAoECs invasion. (B) Invasion length of HAoECs. (C) Invasion number of HAoECs. Scale bar 100 µm.

The effect of DMSA-TiO_2_ on HAoECs tube development was further evaluated. Both the length of the tubules and the development of branch points in the presence of DMSA-TiO_2_ are considerably hindered by the capillary tubes (Figure 6(B,C)). Furthermore, the treatment of HAoECs with DMSA-TiO_2_ containing a low DMSA-TiO_2_ concentration resulted in successfully reducing the amount of invaded cells relative to untreated, followed by Transwell *in-vitro* assay (Figure 6(A)).

### 3.6. Outcomes of chick chorioallantoic membrane

**Figure 7.**
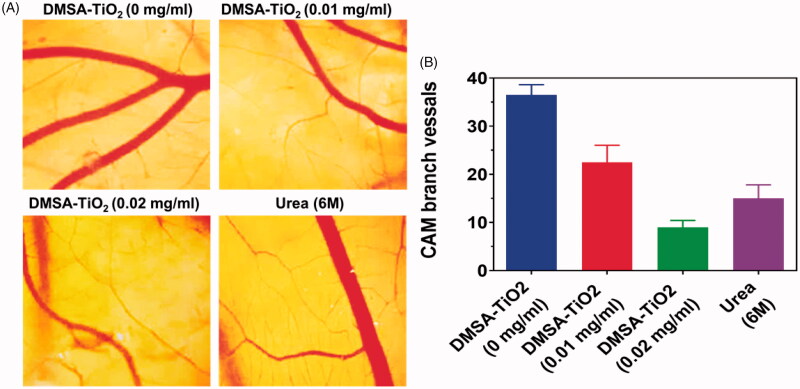
(A) Representative images of chicken chorioallantoic membrane (CAM) vessels. Angiogenesis inhibition by DMSA-TiO_2_ in a model of CAM. Fertilized eggs were incubated and humidified for 8 days before *ex vivo* cultures. Then, sterile filter paper soaked (0.01 and 0.02 mg/ml) and Urea 6 M coated the embryos for 24 h. Saline was comprised as references. (B) The branch number of chicken chorioallantoic membrane (CAM) vessels. Scale bar 1 mm.

Based on these *in vitro* findings, the angiogenesis regulation *in vivo* using this DMSA-TiO_2_ was further examined (Guo et al., 2016; Ma et al., 2017; Winter et al., 2018). The dense capillary network of chick embryo chorioallantoic membrane (CAM) was therefore commonly used to identify angiogenic factors and evaluate the antiangiogenic action of an extensive array of compounds during development. The sterile filter paper containers 1, 2, and 3 in DMSA-TiO_2_ were used to cultivate fertilized CAM tissues on day 8 of embryo development. Figure 7(A) showed the findings. The CAM tissue was thick and vascular structures formed in space after treatment with saline containing filter papers. In direct contrast to DMSA-TiO_2_ therapy, vascular network development in fertilized eggs was significantly inhibited, and this inhibitory influence was comparable to that of the commercially important drug. The number of branched vessels in each community was also quantitated for the development of the blood vessel (Figure 7(B)). The effect of DMSA-TiO_2_ (0.01 and 0.02 mg/ml) and along with Urea 6 M on angiogenesis *in vivo* inhibitors was further confirmed. These CAM findings thus explicitly affirm the pharmacological effects of the easily assembled DMSA-TiO_2_.

### 3.7. Toxicity of DMSA-TiO_2_

**Figure 8.**
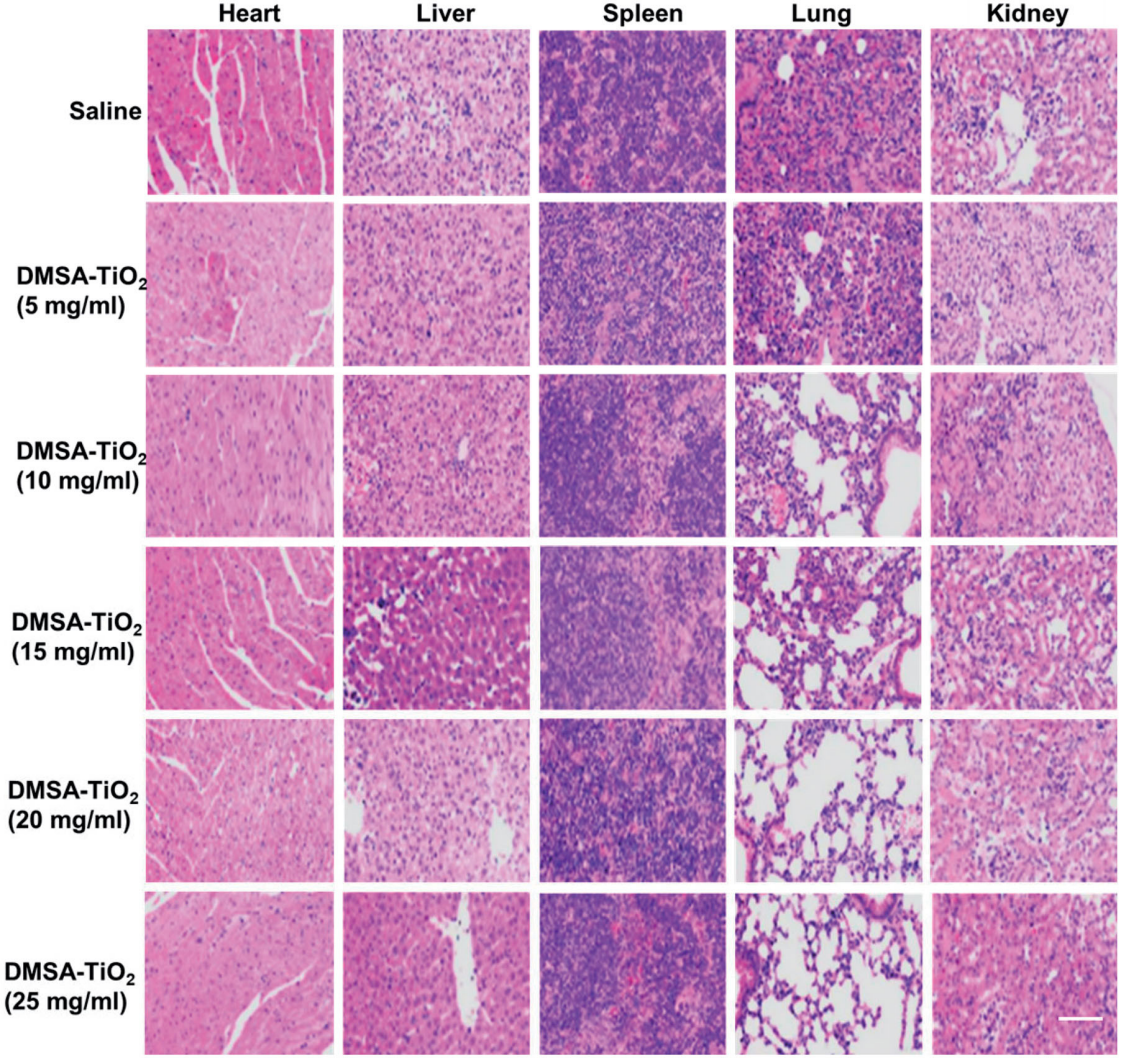
H&E staining of organs sections including heart, liver, spleen, lung, and kidney in each group. Scale bar 100 μm.

For 14 days, we have treated mice at various doses of DMSA-TiO_2_ and find no difference in body weight gains for classes of different dose-treated mice. The organ/body weight ratio for the liver, reindeer, spleen, lung, and heart of mice after i.p. exposure for 14 days did not improve with the low dose of DMSA-TiO_2_ (5 and 10 mg kg^−1^) (Figure 8). Saline was used as a control group. However, the large doses of DMSA-TiO_2_ (15, 20, and 25 mg kg^−1^) substantially increased the liver, lung, kidney, spleen, and heart organs ratio in mice (Figure 8). No blood biochemistry index changes were recorded at lower doses (15, 20, and 25 mg kg^−1^) of DMSA-TiO_2_ (Figure 8).

**Figure 9.**
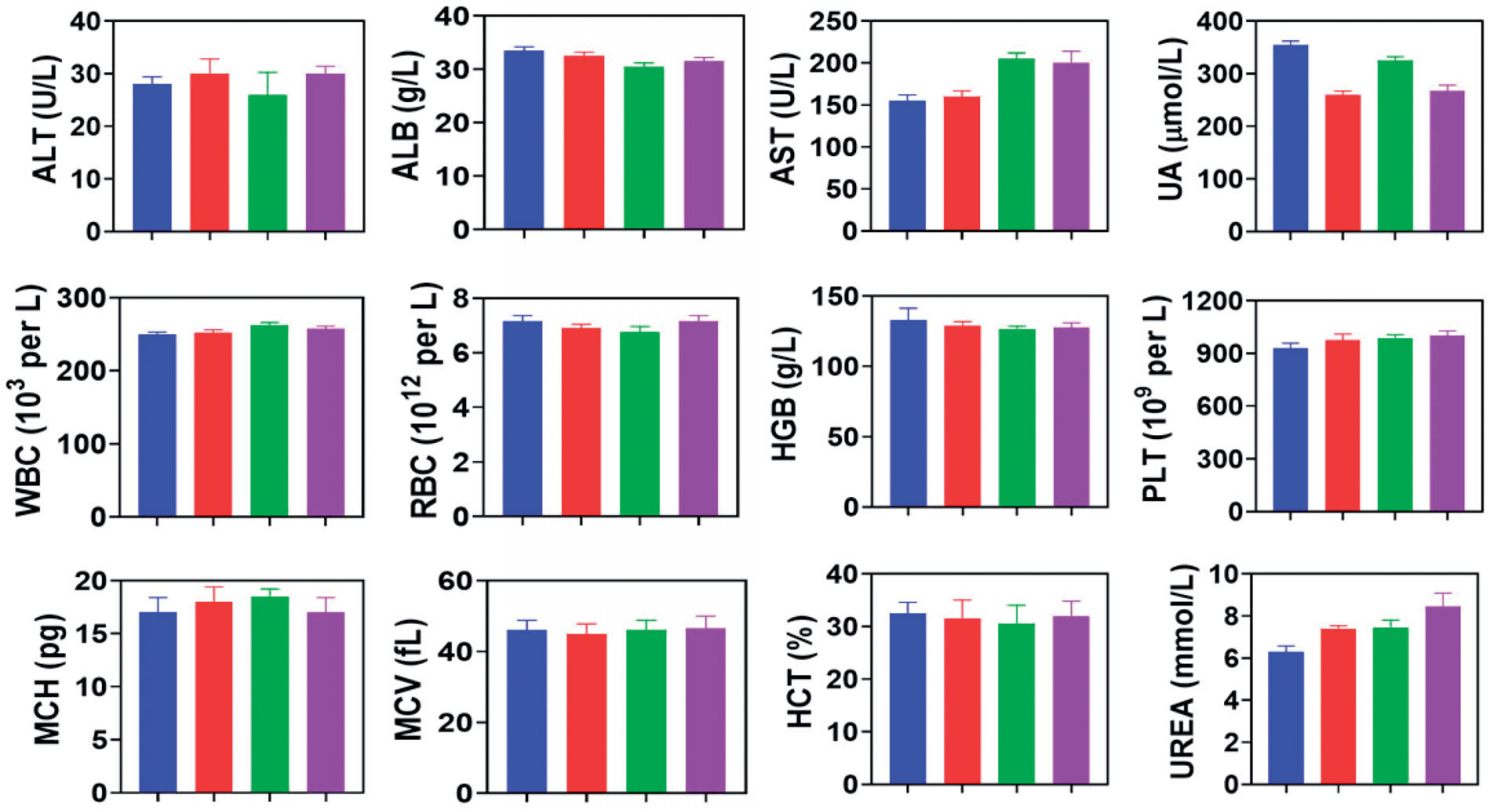
Biochemical parameter in serum were detected in various groups after different treatment. Saline (blue), DMSA-TiO_2_ (15 mg/ml) (Red), DMSA-TiO_2_ (20 mg/ml) (green), and DMSA-TiO_2_ (25 mg/kg) (violet).

Serum biochemistry profiles including alanine aminotransferase (ALT), albumin (ALB), aspartate aminotransferase (AST), the ratio of globulin and albumin (G/A), white blood cells (WBC), red blood cells (RBC), platelets (PLT), mean platelet volume (MPV), mean corpuscular volume (MCV), mean corpuscular hemoglobin concentration (MCHC), mean corpuscular hemoglobin (MCH), hemoglobin (HGB), hematocrit (HCT), blood urea nitrogen (BUN) levels showed no apparent injury and distinct interference of the physiological regulation by the DMSA-TiO_2_. Furthermore, no apparent damage or inflammatory lesions were detected in the H&E-stained pathological sections of major organs (kidney, lung spleen, liver, and heart) from the sacrificed mice (Figure 9).

## 4. Conclusions

To conclude, this analysis reveals the possibility of causing dose-dependence cytotoxic events for DMSA-TiO_2_ nanoparticles accumulated by the HAoECs. HAoECs subjected to even a little DMSA-TiO_2_ can be affected without apparent cell toxicity by endocrine activity and angiogenic functions. The angiogenesis results reveal that the DMSA-TiO_2_ nanomaterials potentially inhibit the HAoECs. Further, the results of toxicity examination in animal models demonstrate the superior activity in the DMSA-TiO_2_ with different formulations. Before using them in medicine, therefore, careful appraisal of DMSA-TiO_2_ nanomaterials *in vivo* is essential.
